# Efficacy and Safety of Acupuncture in the Treatment of Radicular Cervical Spondylosis: A Systematic Review and Meta-Analysis

**DOI:** 10.2174/0113862073265007231108050338

**Published:** 2023-11-08

**Authors:** Hongfei Zhao, Congan Wang, Xuan Wang, Jing Ju, Chunchun Yan, Bin Shi

**Affiliations:** 1 School of Acupuncture-Tuina, Shandong University of Traditional Chinese Medicine, Jinan 250013, China;; 2 Shandong First Medical University Affiliated Neck Shoulder Waist Leg Pain Hospital, Jinan, 250031, China;; 3 Postdoctoral Mobile Station of Shandong University of Traditional Chinese Medicine, Jinan, 250399, China;; 4 Weihai Hospital of Traditional Chinese Medicine, Shandong, 264200, China

**Keywords:** Cervical spondylotic radiculopathy, randomized controlled trials, acupuncture treatment, traction therapy, safety of acupuncture, pain index

## Abstract

**Background:**

Cervical spondylotic radiculopathy is a serious and common degenerative disease of the cervical spine due to irritation and compression of the nerve roots of the cervical spine, resulting in a series of clinical symptoms based on sensory, motor and reflex disorders, such as numbness and pain in the neck, shoulders, upper limbs and fingers. Acupuncture is highly effective in treating CSR and has become a common treatment accepted by patients. This study aims to systematically review and analyze existing randomized controlled trials (RCTs) to evaluate the efficacy and safety of acupuncture in the treatment of CSR.

**Methods:**

We used the following eight databases for literature data search: PubMed, EMBASE, The Cochrane Library, Web of Science, China National Knowledge Infrastructure, China Biology Medicine Disc (CBMdisc), Wanfang Database and China Science and Technology Journal Database (VIP). The search consisted of randomized controlled studies of acupuncture for CSR between 2000 and 2020 and the methodological quality of the included studies was assessed according to the Cochrane Collaboration's “Risk of Bias Assessment Tool.”RevMan 5.4 software was used for statistical analysis only. Study screening, data extraction and statistics, and assessment of the risk of bias of the included studies were performed independently by two reviewers.

**Results:**

27 studies with 3124 patients were included. The results of the meta-analysis of the total efficiency index for acupuncture for CSR were [RR = 1.14,95% CI (1.09,1.19)]. The results of the meta-analysis of the PPI index were [MD = -0.35, 95% CI (-0.61,-0. 09)]. The results of META analysis of the total effective rate, VAS score, PRI(A) score, PRI(S) score and PRI(T) score showed heterogeneity in the studies included for each outcome index, and sources of heterogeneity were sought through subgroup analysis and sensitivity analysis to ensure more stable and reliable data results. The results of the combined meta-analysis showed that the treatment group was significantly more effective than the control group and more effective in lowering the nerves to reduce the pain index in patients with CSR, with a statistically significant difference (*P*<0.05). This indicates that acupuncture treatment is superior to traction for CSR.

**Conclusion:**

Acupuncture is significantly more effective than traction therapy in the treatment of cervical spondylosis and can reduce the pain index of patients with CSR.

## 1 INTRODUCTION

Cervical spondylotic radiculopathy (CSR) is a common type of cervical spondylosis involving a pathological process of the cervical nerve roots caused by compression or inflammation of the nerve roots [1]. The most frequent level of nerve root compression is C7, followed by C6. It is caused by degenerative changes in the cervical spine. Patients may present with pain or numbness in the neck, back of the shoulder, and radiation to the distal upper extremities, such as in the triceps, dorsolateral forearm, and dorsum of the long thumb [2]. As the growth the age, within the disc nucleus pulposus chemical composition and fiber ring changes, leading to its degeneration can be compressed, elastic will be smaller, intervertebral disc's ability to retain moisture is also reduced, so the disc will be side to bump into the spinal canal. Small yellow ligaments and joint capsules can push backward fold extrusion, increasing the size of the spinal canal and vertebral foramen narrow, thus oppression adjacent nerve roots [3]. The affected nerve roots often experience some degree of pain, numbness, loss of sensation, and altered reflexes in the innervation area. The world epidemiological survey showed that the incidence of the disease was 107.3/100000 in males and 63.5/100000 in females, and the high incidence period of the disease was between 50 and 54 years old [4].

At present, there are many clinical works of literature about acupuncture in the treatment of CSR, but there are few randomized controlled trials with a large sample size. Further investigation into the clinical efficacy and safety of acupuncture in the treatment of CSR, we used the evidence-based medicine method to collect data published from January 2000 to January 2022 on acupuncture therapy treatment in clinical randomized controlled trials of CSR. This study is intended to evaluate the efficacy and safety of needles for CSR by systematically reviewing and analyzing the currently available randomized controlled trials (RCTs), which will provide evidence support for clinical decision-making.

## 2 MATERIALS AND METHODS

### 2.1 Study Registration

This protocol was registered with the International Platform of Registered Systematic Review and Meta-Analysis Protocols (INPLASY) on 02 September 2022 and was last updated on 02 September 2022 (registration number INPLASY202290007) [5]. And has been presented in the in plastic PROTOCOL. The PRISMA guidelines [6] and the recommendations of the Cochrane Handbook for Systematic Reviews of Interventions were complied with this systematic review and meta-analysis.

### 2.2 Data Sources And Search Strategy

Search strategies included subjects (patients with CSR), interventions (application of acupuncture, electroacupuncture, acupuncture combined with massage, acupuncture combined with massage therapy and traction in the treatment group, and traction therapy in the control group) and study type (RCTs).

We used a combination of keyword search and free-word search to search the following eight electronic databases for eligible studies published between January 1, 2000, and January 1, 2022: PubMed, EMBASE, The Cochrane Library, Web of Science, China National Knowledge Infrastructure, China Biology Medicine disc (CBMdisc), Wanfang Database and China Science and Technology Journal Database(VIP). Search terms in English and Chinese include 'spondylitis', 'cervical spondylosis', 'acupuncture', 'randomized controlled trial,' 'acupuncture therapy' and many other synonyms or near-synonyms. Take the Pubmed database retrieval scheme as an example. The PubMed search strategy is summarised in Table **1**. The search strategy will be modified according to the needs of other electronic databases.

### 2.3 Research Methods

Reference to the “*Expert consensus on the standardization of diagnosis and treatment of cervical spondylotic radiculopathy*” [7] included patients with CSR who met the following diagnostic criteria (age and sex not restricted):①There was localized radicular compression.②Positive brachial plexus pull test or neck compression test.③X-ray, CT or MRI showed hyperostosis or osteophytes around the nerve roots or foraminal stenosis caused by cervical degenerative disease. ④excluding scapulohumeral periarthritis, thoracic outlet syndrome and other diseases, mainly with upper limb pain.

Reproducible studies were excluded. Two types of randomized controlled trials were included: The patients in the experimental group were treated with single acupuncture or acupuncture combined with traction therapy, but the patients in the control group were treated with traction therapy.

The outcome indicators were the effective rate of acupuncture in the treatment of CSR, McGill Pain Questionnaire (MPQ), visual analogue scale (VAS). The pain intensity, physical function improvement, social function score, overall health status, and the safety of acupuncture were measured and evaluated. Data were extracted using predesigned forms that extracted clinical characteristics (subjects, interventions, and outcome measures), treatment details, methodological characteristics, and outcomes for each study.

We assessed the quality and risk of bias of all included articles using the Cochrane recommended risk of bias assessment tool. The Jadad rating scale was chosen for methodological quality assessment, with two evaluators assessing the quality of all included studies on three components: randomisation, blinding, and withdrawal or follow-up, with the three components set on a scale of 0-2 according to the evaluation criteria and a total score of 0-7 for the three components. Two trained evaluators each read the full text independently and then reviewed it according to the same data extraction form. In case of disagreement, the two evaluators discussed and reached agreement or a third evaluator assisted in resolving the disagreement.

Meta-analysis was performed on all included studies using RevMan 5.4 software. Risk ratios (RR) were used for dichotomous variables and mean differences (MD) were used as effect indicators for continuous variables, both using 95% confidence intervals (95% CI). Heterogeneity was tested, and if *P*>0.1 and I2<50%, heterogeneity was considered small, and a fixed-effects model was used; otherwise, a random-effects model was used, and sensitivity analyses were performed to explore the sources of heterogeneity, with subgroup analyses performed if necessary. The results of the data analysis are presented as forest plots and funnel plots.

Study screening was carried out by two researchers by reading the title, and abstract and excluding apparently irrelevant and duplicate literature. For repeat screening, the two researchers cross-checked, downloaded and read the full text, and finalised the inclusion of the study based on the nadir criteria. In case of disagreement, the agreement was reached by discussion between the two parties or through the assistance of a third party.

Data extraction was carried out independently by the two evaluators according to a pre-designed form, which included: ①authors and year of publication; ②basic information: number of people in each group and comparable status at baseline (*e.g.,* gender, age); ③different treatment measures taken in the treatment and control groups and the duration of treatment required; and ④study outcomes: outcome indicators required for outcome evaluation.

## 3 RESULTS

### 3.1 Literature Search Results

Import the documents retrieved from the various databases into NoteExpress 3.2.0 software. A total of 14,649 potentially relevant studies were identified through a database search. After the literature review, 5112 duplicate studies were excluded, 6860 studies were excluded by reading titles and abstracts, and 2650 studies were excluded based on inclusion and exclusion criteria. Finally, 27 literatures were included after excluding non-randomized controlled experiments and other literatures that did not meet the research needs [8-34]. See Fig. (**1**) for the literature search process.

A total of 3124 patients were included in the study, including 1426 in the treatment group (including 342 patients who received manual acupuncture, 409 patients who received electroacupuncture and 675 patients who received combination therapy) and 1326 in the control group (patients who received traction or combination therapy). Information on the basic characteristics of the included studies is detailed in Table **2**.

### 3.2 Assessment of Risk of Bias

Each study reported randomization of patients into experimental and control groups. Only four studies [8, 9, 13, 21] did not provide detailed information about the randomization process. One [20] study conducted a blind analysis of outcome evaluators. Concerning selective reporting bias, we judged that all trials represented the expected results. The risk of bias in the included studies is shown in Fig. (**2**).

### 3.3 Meta-analysis Results

#### 3.3.1 Total Effective Rate

**Fig. (3):**
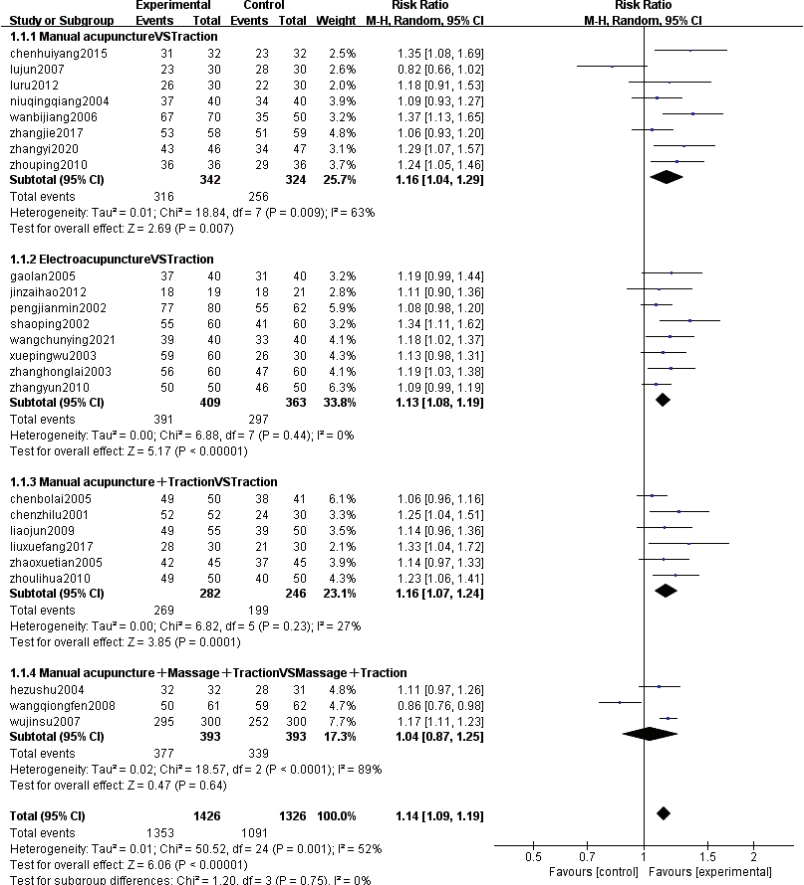
Forest plots for subgroup analysis of the total efficiency index (dichotomous variables) for the 25 included studies.

For the research question of the efficiency of acupuncture in the treatment of CSR compared to traction therapy, the total clinical efficiency was selected as the outcome indicator and meta-analysis was performed on the 25 [10-34] included studies. These 25 studies were categorized into 4 subgroups based on the method of intervention. Of these, 8 studies [10-17] were included in the manual acupuncture versus traction group, the results of meta-analysis showed that the combined sample size was 572 cases and the combined effect was statistically significant [MD=1.16, 95% CI (1.04,1.29), Z=2.69, *P*=0.00^7^],this suggests that the overall efficacy of manual acupuncture in the treatment of neurogenic cervical spondylosis is superior to traction. 8 studies [18-22, 26-28] were included in the electroacupuncture *versus* traction group, the results of meta-analysis showed that the combined sample size was 688 cases, and the combined effect was statistically significant [MD=1.13, 95% CI (1.08,1.19), Z=5.17, *P*<0.0000^1^]. This suggests that the overall efficacy of electroacupuncture is superior to traction. 6 studies [23-25, 29-31] were included in the manual acupuncture combined with traction versus traction group, this meta-analysis showed that the combined sample size was 468 cases and the combined effect was statistically significant [MD=1.16, 95% CI (1.07,1.24), Z=3.85, *P*=0.000^1^]. This suggests that the efficacy of acupuncture combined with traction is superior to that of single traction. 3 studies [32-34] were included in the manual acupuncture combined with nudging and traction versus nudging combined with traction group.This meta-analysis showed that the combined sample size was 716 cases and the combined effect was not statistically significant [MD=1.14, 95% CI (1.09,1.19), Z=0.47, *P*=0.64]. The inclusion of only three studies and the larger sample size in this group compared with the results of the other three subgroups may be the main reason for the lack of statistical significance. Overall Meta-analysis results showed, less heterogeneity in statistical results (*P*=0.001, I^2^=52%) and acceptable clinical heterogeneity, and a random effects model was used for the Meta-analysis. The results showed a combined sample size of 2752 cases and a statistically significant combined effect [RR=1.14, 95% CI(1.09,1.19)], Z=6.06, *P*<0.00001. The small diamond-shaped square was located to the right of the null line, *i.e.,* favouring the acupuncture treatment group, and the results did not intersect with the null line in all but one study. This implies that acupuncture has better efficacy in treating CSR compared to traction therapy (Fig. **3**).

**Fig. (4):**
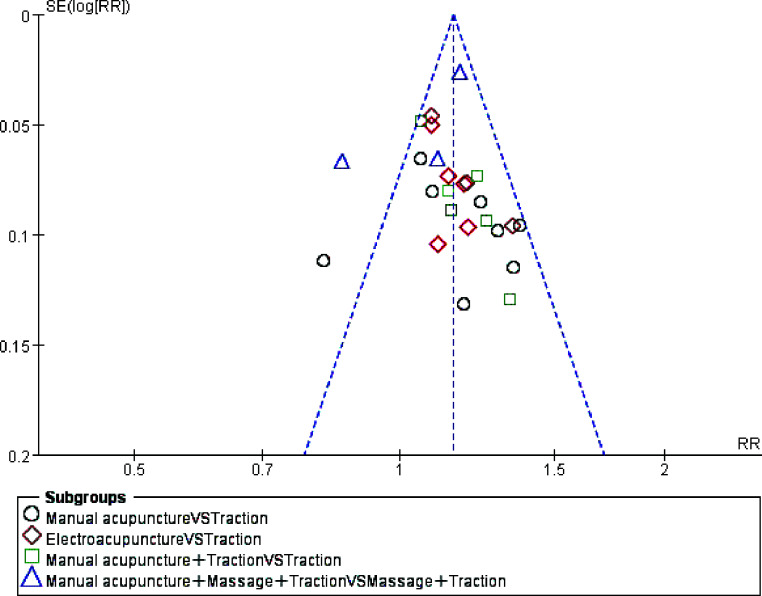
Meta-analysis funnel plot of total efficiency scores (dichotomous variables) for the 25 included studies.

According to the requirements of the funnel plot, more than 10 papers needed to be included, so the 25 dichotomous data of the total efficiency index were analysed in a biased funnel plot according to the outcome indicators (Fig. **4**). Based on the funnel plot analysis, most were within the 95% confidence interval and roughly symmetrical from left to right, but unevenly distributed at the top and bottom, with individual studies outside the 95% confidence interval, the results showed some publication bias and it is thought that this may be due to the uneven quality of the literature included in the studies.

#### 3.3.2 VAS Score

For the research question on the improvement of pain in patients with CSR with acupuncture treatment compared to traction therapy, the clinical VAS score was chosen as the outcome indicator and Meta-analysis was also conducted on data from the 8 [8, 11, 14, 17, 23-25, 32] continuous-type variables included. These 8 studies were categorized into two subgroups based on the method of intervention, with 4 studies [8, 11, 14, 17] included in the manual needling versus traction group and the remaining 4 studies [23-25, 32] included in the manual needling combined with traction versus traction group. The meta-analysis of the manual needling versus traction group showed a combined sample size of 361 cases with a statistically significant combined effect [MD=-1.09, 95% CI (-1.94,-0.25), Z=2.55, *P*=0.01]. This suggests that manual needling is more effective. The results of manual needling combined with traction compared to the traction group showed a combined sample size of 382 cases, and the combined effect was not statistically significant [MD=-1.01, 95% CI (-2.39,0.36), Z=1.44, *P*=0.15]. According to the forest plot, it was found that this study of wang qiong fen 2008 [32] was on the right side of the null line, which should be the main reason for the non-statistically significant results, closely related to its maximum sample size.

**Fig. (6):**
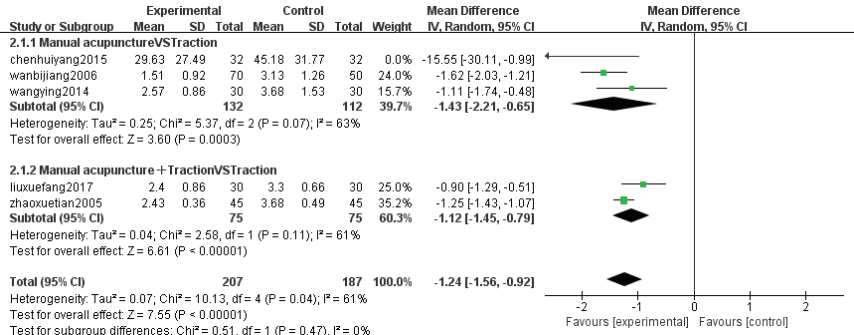
VAS score sensitivity analysis forest diagram.

The statistical results showed a large heterogeneity (*P*<0.00001,I2=97%) and Meta- analysis was performed using a random effects model. The results showed that the combined sample size was 743 cases and the combined effect was not statistically significant [MD=-1.08, 95%CI(-1.95,-0.20), Z=2.41, *P*=0.02] Fig. (**5**). The small diamond shaped square was located to the left of the null line, *i.e.* favouring the acupuncture treatment group, and the results did not intersect with the null line in all but one study. This implies that acupuncture treatment improves pain symptoms in patients with CSR more than traction therapy. Sensitivity analyses were performed as the VAS index showed greater heterogeneity after subgroup analysis. The consistency and quality of results were assessed by deleting each study individually. The results showed a decrease in heterogeneity after removing 3 [14, 25, 32] studies (x2=10.13, I2=61%, *P*=0.04), therefore these 3 studies were considered to be the source of heterogeneity and the reason for the large heterogeneity could be the large variation in the sample size of the literature and the uneven quality of the literature (Fig. **6**).

#### 3.3.3 McGill Pain Questionnaire

**Fig. (7):**
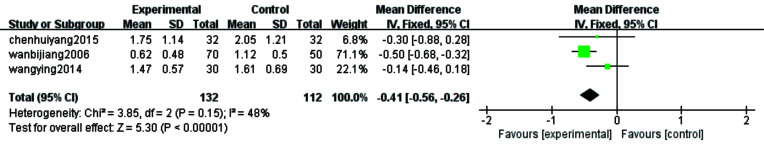
Meta-analysis forest plots of PPI scores (continuous type variables) for the three included studies.

The three [8, 11, 17] papers involved the McGill PPI index, all with manual needling versus traction therapy, included a total of 244 patients. The data were all continuous variables with low heterogeneity (I2=48%, *P*=0.15), showing that [MD = -0.41, 95% CI(-0.56, -0.26), Z=5.30,*P<*0.00001] (Fig. **7**). The results were statistically significant, indicating that the efficacy of manual needling group in improving patients' pain symptoms was better than traction.

**Fig. (8):**
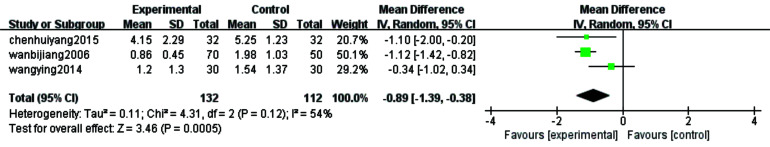
Meta-analysis forest plots of PRI(A) scores (continuous type variables) for the three included studies.

**Fig. (9):**
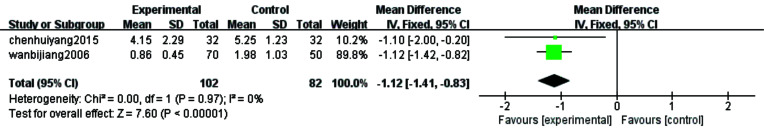
PRI(A) score sensitivity analysis forest diagram.

Three [8, 11, 17] papers involved the McGill PRI(A) index with low heterogeneity (I2=54%, *P*=0.12) and the combined effect size results showed [MD=-0.89, 95% CI (-1.39,-0.38), Z=3.46,*P*=0.0005] (Fig. **8**). Heterogeneity was reduced by performing a sensitivity analysis on the results, excluding each one individually and then performing a Meta-analysis (I2=0%, *P*=0.97). It is presumed that this excluded literature [8] was the source of heterogeneity (Fig. **9**). This result is statistically significant and suggests that manual needling is more efficacious in improving the sensory item pain index.

**Fig. (10):**
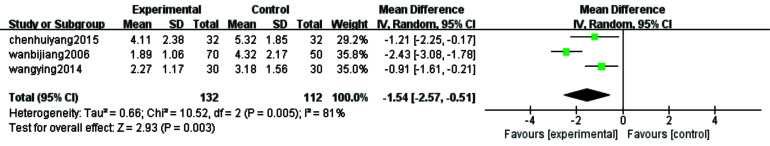
Meta-analysis forest plots of PRI(S) scores (continuous type variables) for the three included studies.

**Fig. (11):**
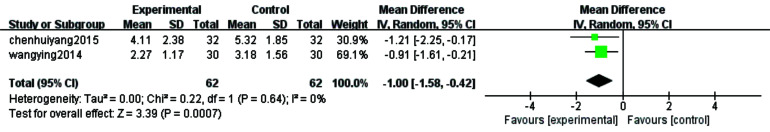
PRI(S) score sensitivity analysis forest diagram.

Three [8, 11, 17] papers involved the McGill PRI(S) index, with heterogeneity between studies (I2=81%, *P*=0.005), and the combined effect sizes showed MD=-1.54, 95% CI [-2.57, -0.51], Z=2.93, *P*=0.003 (Fig. **10**). Sensitivity analysis was performed on the results, excluding each one before performing Meta-analysis, heterogeneity was reduced (I2=0%, *P*=0.64). Presumably, the excluded literature [17] was a heterogeneous source (Fig. **11**). The results of the study were statistically significant and indicated greater efficacy of manual needling.

**Fig. (12):**
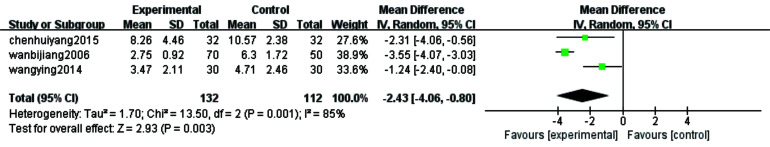
Meta-analysis forest plots of PRI(T) scores (continuous type variables) for the three included studies

**Fig. (13):**
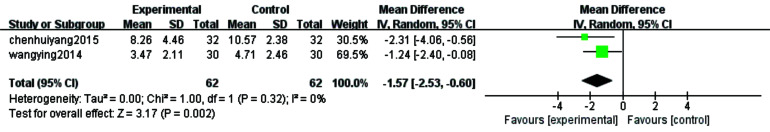
Forest plot for sensitivity analysis of PRI(T) scores.

Three [8,11,17] papers involved the McGill PRI(T) index, with high heterogeneity between studies (I2=85%, *P*=0.32) and a combined effect size of [MD=-2.43, 95% CI (-4.06, -0.80), Z=2.93, *P*=0.003] (Fig. **12**). A sensitivity analysis was performed on the results, and heterogeneity was reduced by excluding each piece of literature prior to Meta-analysis (I2=0%, *P*=0.32). It was hypothesized that the excluded literature [17] was the source of heterogeneity (Fig. **13**). This result was statistically significant and suggests that the efficacy was more significant in the manual needling group.

The results of the above Meta-analysis showed that the analysis of the PPI index and PRI index in the McGill scale scores indicated that the treatment group scored lower than the control group, which indicated that acupuncture therapy was more effective than traction therapy in improving pain in patients with CSR.

### 3.4 Safety Analysis of Acupuncture

Four studies [9, 12, 13, 19] have analysed the safety of acupuncture in the treatment of cervical spondylosis. One study [13] reported three cases of acupuncture sickness reactions and others reported minor pain and bleeding at the needle holes, all without adverse effects on normal vital signs.

### 3.5 Sensitivity Analysis

Sensitivity analysis was carried out by changing the statistical analysis model of pooled effect size. The results showed that after changing the statistical analysis model, the combined effect size of each outcome index did not change significantly, and had statistical significance, indicating that the results were stable and reliable Table **3**.

## 4 DISCUSSION

CSR refers to cervical disc degeneration, cervical bone hyperplasia, and cervical joints cervical nerve roots and other tissues are stimulated or compressed in the spinal canal or foramina due to ligament loosening and dislocation [35] leading to a certain degree of pain, numbness, loss of sensation and reflex changes in the innervation area [36].Modern medical studies have shown that the anatomical and pathological manifestation of CSR is compression of the cervical nerve root, which is related to stimulation of inflammatory factors [37]. According to traditional Chinese medicine, CSR is mainly caused by tension, trauma, wind, cold, dampness, muscle spasm and poor posture, [35] which belongs to the category of cervical pain, *etc*., and its pathological basis is neck strain, liver and kidney deficiency, Qi and blood deficiency, and loss of nutrition of muscles and veins. Acupuncture as a common therapy for CSR is safe and effective. Acupuncture can improve the nutritional environment and blood circulation of neck tissue, thus accelerating local metabolism, reducing nerve root edema, promoting inflammation absorption, reducing pain, and maintaining cervical spine stability [38]. In recent years, many scholars have refined acupuncture therapy and applied more traditional acupuncture methods to the treatment of CSR. Abdominal acupuncture therapy by regulating the body balance of Yin and Yang to achieve the purpose of channeling meridian, activating collateral, and relieving pain [39]. Balance needle is a kind of acupuncture therapy guided by the combination of Yin and Yang theory of traditional Chinese medicine and the neuroregulation theory of Western medicine. Most of its acupoints are distributed around the nerve to achieve the purpose of rapid activation of the central nervous system for self-regulation to relieve pain [40]. The Fire needle is fast in and out to stimulate human Yang Qi, stimulate the qi of the meridians. Water acupuncture, also known as acupoint injection therapy, injects drugs into acupoints through syringes to maximize the combined effect of acupuncture and drugs. In addition, “moxibustion” is also one of the commonly used therapies. Warm acupuncture is a better combination of “needle” and “moxibustion”, and heat-sensitive moxibustion is an alternative acupuncture therapy that stimulates qi transmission through moxibustion of heat-sensitive points with moxa sticks [41]. In summary, there are numerous acupuncture therapies, based on the guiding principle of identification and treatment characteristic of traditional Chinese medicine, which, combined with the results of this study, showed that manual acupuncture, electroacupuncture, or therapies combined with tuina and traction were superior to traditional traction, both in terms of improving the patients' pain symptoms and the occurrence of adverse effects. However, analysis of the risk of bias in the 27 included studies showed the following problems: the quality of included studies was uneven and some studies did not describe the concealment and blinding of the distribution protocol. Acupuncture point selection and number of acupuncture points varied among the included literature, the depth and intensity of acupuncture were not described, and the studies were not sufficiently stable. In future studies in the literature, we will pay more attention to the refinement of acupuncture therapies, explore more efficient and safe traditional acupuncture therapies, and expand the clinical treatment ideas of CSR.

## CONCLUSION

In this paper, the main interventions used were acupuncture, electro-acupuncture, combined tui-na and traction, while the control group used traction therapy only. The results of the Meta-analysis showed that acupuncture, electro-acupuncture and combined therapy were more effective than traction therapy in the treatment of CSR. Patients with CSR treated with acupuncture had lower pain scores, physical function scores, social function scores and overall health scores than those treated with traction, and the differences were statistically significant.

## Figures and Tables

**Fig. (1):**
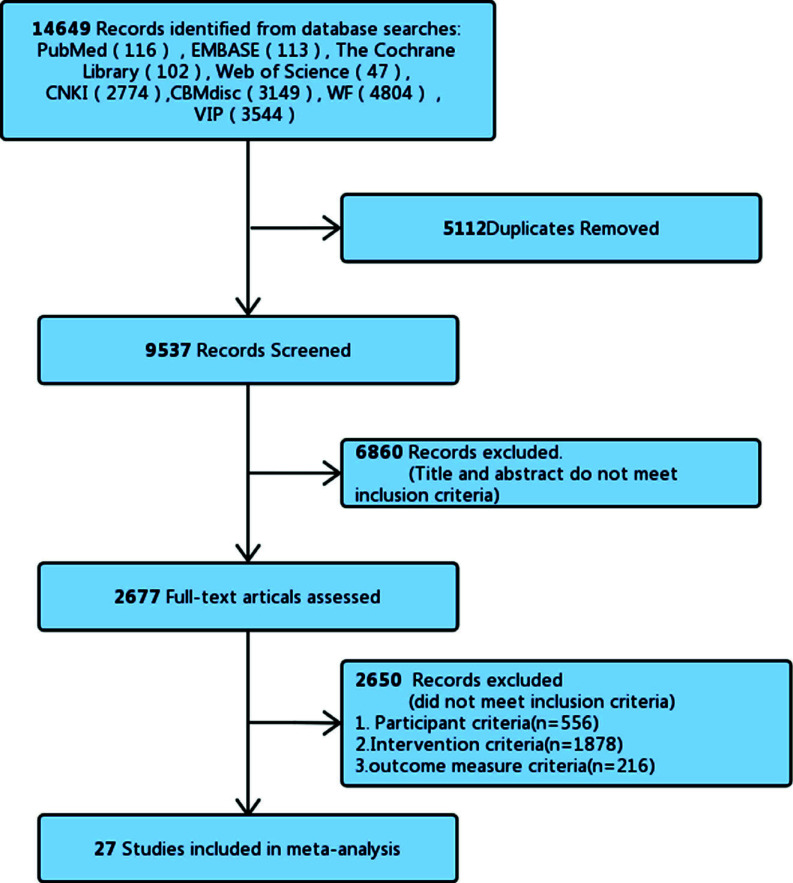
Flow chart for literature search related to acupuncture for CSR.

**Fig. (2):**
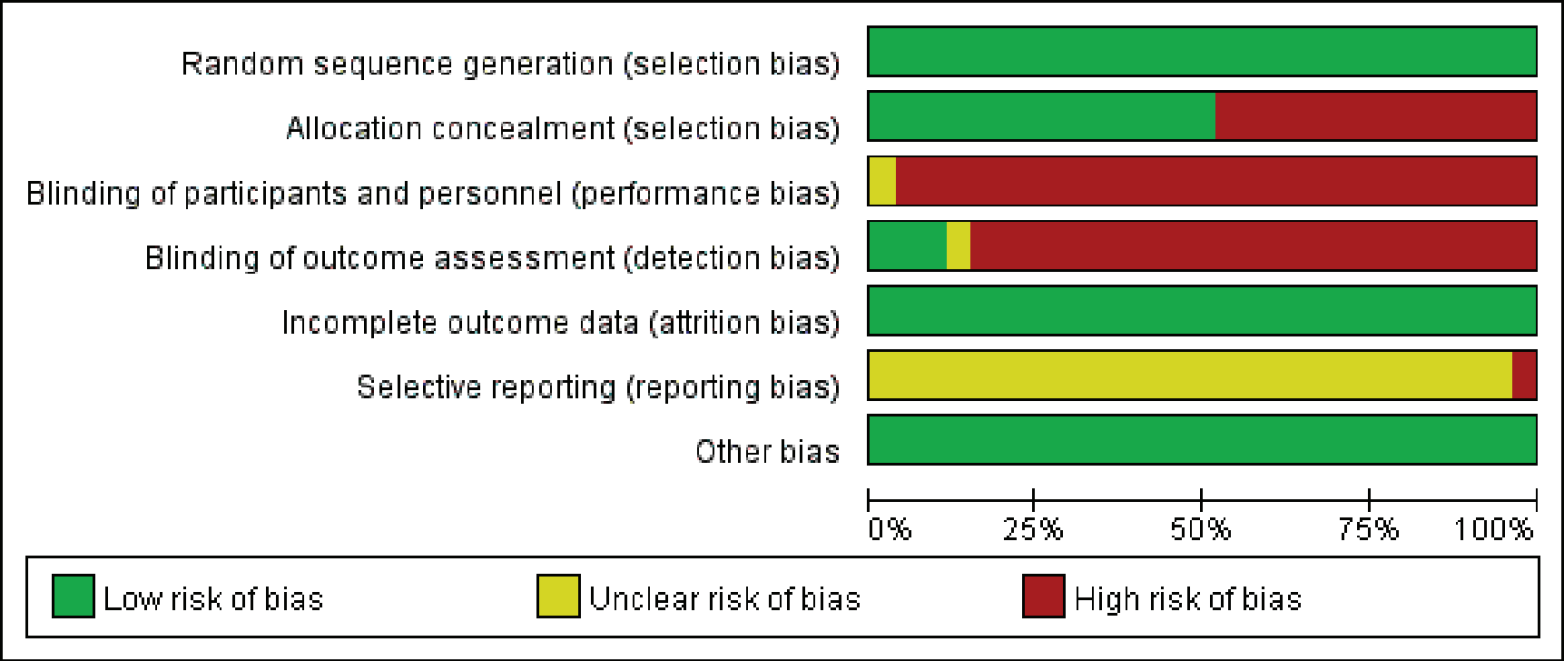
Assessment of risk of bias. Green circles indicate a low risk of bias, yellow circles indicate an unclear risk of bias, and red circles indicate a high risk of bias.

**Fig. (5):**
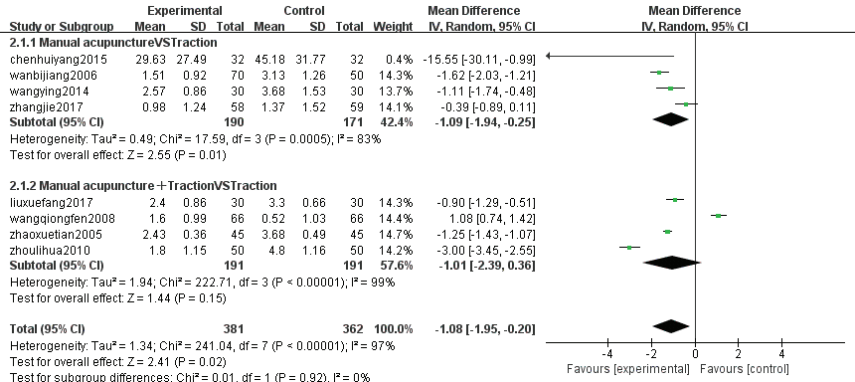
Forest plots for subgroup analysis of VAS scores (continuous type variables) for the eight included studies.

**Table 1:** PubMed search strategy.

**Search Strategy used in PubMed Database.**	
**Number**	**Search Terms**
1	“Spondylosis”[Mesh]
2	Nervous Cervical Type Cervical Spondylosis[Title/Abstract] OR cervical spondylotic radiculopathy[Title/Abstract] OR nerve root cervical Spondylopathy[Title/Abstract] OR nerve root cervical spondylosis[Title/Abstract] OR nerve-root cervical spondylosis[Title/Abstract]
3	“Acupuncture”[Mesh]
4	Acupuncture therapy[Title/Abstract] OR Acupuncture needle[Title/Abstract] OR Manual acupuncture[Title/Abstract] OR Electroacupuncture[Title/Abstract] OR Needling[Title/Abstract])
5	Randomized Controlled Trials as Topic”[Mesh]
6	Randomized controlled[Title/Abstract] OR clinical trial[Title/Abstract] OR Randomized[Title/Abstract] OR Randomly[Title/Abstract] OR Trial[Title/Abstract]
7	1 OR 2
8	3 OR 4
9	5 OR 6
10	7, 8 AND 9

**Table 2:** Table of basic characteristics of the included studies. (E is the treatment group, C is the control group).

**Study**	**Cases**		**Course (d)**	**Intervening Cause**		**Evaluating Indicator**
	**E**	**C**		**E**	**C**	
Wang Ying2014 [8]	30	30	14	acupuncture	traction	VAS,PRI-A,PRI-S,PRI-T,PPI
Bo Zhiyun 2005 [9]	150	150	10	acupuncture	traction	Adverse reactions
Zhang Yi 2020 [10]	46	47	10	acupuncture	traction	Total effective rate
Chen Huiyang2015 [11]	32	32	20	acupuncture	traction	Total effective rate, VAS, PRI-A, PRI-S,PRI-T, PPI
Niu Qingqiang2004 [12]	40	40	10	acupuncture	traction	Total effective rate,Adverse reactions
Lu Ru 2012 [13]	30	30	14	acupuncture	traction	Total effective rate,Adverse reactions
Zhang Jie 2017 [14]	60	60	10	acupuncture	traction	Total effective rate,VAS
Lu Jun 2007 [15]	30	30	30	acupuncture	traction	Total effective rate
Zhou Ping2010 [16]	36	36	35	acupuncture	traction	Total effective rate
Wan Bijiang2006 [17]	70	50	14	acupuncture	traction	Total effective rate, VAS, PRI-A, PRI-S, PRI-T, PPI
Zhang Honglai 2003 [18]	60	60	45	electropuncture	traction	Total effective rate
Jin Zaihao 2012 [19]	19	21	21	electropuncture	traction	Total effective rate
Wang Chunying 2021 [20]	40	40	28	electropuncture	traction	Total effective rate
Zhang Yun 2010 [21]	50	50	20	electropuncture	traction	Total effective rate
Shao Ping 2002 [22]	60	60	40	electropuncture	traction	Total effective rate
Zhao Xuetian 2005 [23]	45	45	20	acupuncture+traction	traction	Total effective rate,VAS
Liu Xuefang 2017 [24]	30	30	10	acupuncture+traction	traction	Total effective rate,VAS
Zhou Lihua 2010 [25]	50	50	14	acupuncture+traction	traction	Total effective rate,VAS
Peng Jianmin 2002 [26]	80	62	40	acupuncture+traction	traction	Total effective rate
Gao Lan 2005 [27]	40	40	20~40	acupuncture+traction	traction	Total effective rate
Xue Pingwu 2003 [28]	60	30	12	acupuncture+traction	traction	Total effective rate
Chen Bolai 2005 [29]	50	41	21	acupuncture+traction	traction	Total effective rate
Chen Zhilu2001 [30]	52	30	10	acupuncture+traction	traction	Total effective rate
Liao Jun 2009 [31]	55	50	20	acupuncture+traction	traction	Total effective rate,Adverse reactions
Wang Qiongfen 2008 [32]	66	66	10	acupuncture+massage+traction	massage+traction	Total effective rate,VAS
Wu Jinsu 2007 [33]	300	300	Not given	acupuncture+massage+traction	massage+traction	Total effective rate
He Zushu 2004 [34]	32	31	20	acupuncture+massage+traction	massage+traction	Total effective rate

**Table 3:** Sensitivity analysis.

Evaluating Indicator	Sensitivity Analysis Model	Combined Effect Size
Total effective rate	Fixed Effects Model	MD=1.15,95%CI[1.12,1.18]
VAS(McGill)	Fixed Effects Model	MD=-1.06,95%CI[-1.19,-0.93]
PPI	Random Effects Model	MD=-0.35,95%CI[-0.61,-0.09]
PRI(A)	Fixed Effects Model	MD=-1.00,95%CI[-1.26,-0.73]
PRI(S)	Fixed Effects Model	MD=-1.63,95%CI[-2.07,-1.20]
PRI(T)	Fixed Effects Model	MD=-2.43,95%CI[-4.06,-0.80]

## Data Availability

Not applicable.

## LIST OF ABBREVIATIONS

CSR: Cervical Spondylotic Radiculopathy
MPQ: McGill Pain Questionnaire
VAS: Visual Analogue Scale
RR: Risk Ratios
MD: Mean Differences

## STANDARD FOR REPORTING

PRISMA guidelines and methodology were followed.

PRISMA checklist is available on the publisher’s website.

## SUPPLEMENTARY MATERIAL

PRISMA checklist is available as supplementary material on the publisher’s website along with the published article. Supplementary material is available on the publisher’s website along with the published article.
